# Why is the processing of global motion impaired in adults with developmental dyslexia?

**DOI:** 10.1016/j.bandc.2016.07.004

**Published:** 2016-10

**Authors:** Richard Johnston, Nicola J. Pitchford, Neil W. Roach, Timothy Ledgeway

**Affiliations:** School of Psychology, University of Nottingham, Nottingham, UK

**Keywords:** Dyslexia, Poor readers, Vision, Integration, Motion, Form

## Abstract

Individuals with dyslexia are purported to have a selective dorsal stream impairment that manifests as a deficit in perceiving visual global motion relative to global form. However, the underlying nature of the visual deficit in readers with dyslexia remains unclear. It may be indicative of a difficulty with motion detection, temporal processing, or any task that necessitates integration of local visual information across multiple dimensions (i.e. both across space and over time). To disentangle these possibilities we administered four diagnostic global motion and global form tasks to a large sample of adult readers (*N* = 106) to characterise their perceptual abilities. Two sets of analyses were conducted. First, to investigate if general reading ability is associated with performance on the visual tasks across the entire sample, a composite reading score was calculated and entered into a series of continuous regression analyses. Next, to investigate if the performance of readers with dyslexia differs from that of good readers on the visual tasks we identified a group of forty-three individuals for whom phonological decoding was specifically impaired, consistent with the dyslexic profile, and compared their performance with that of good readers who did not exhibit a phonemic deficit. Both analyses yielded a similar pattern of results. Consistent with previous research, coherence thresholds of poor readers were elevated on a random-dot global motion task and a spatially one-dimensional (1-D) global motion task, but no difference was found on a static global form task. However, our results extend those of previous studies by demonstrating that poor readers exhibited impaired performance on a temporally-defined global form task, a finding that is difficult to reconcile with the dorsal stream vulnerability hypothesis. This suggests that the visual deficit in developmental dyslexia does not reflect an impairment detecting motion *per se*. It is better characterised as a difficulty processing temporal information, which is exacerbated when local visual cues have to be integrated across multiple (>2) dimensions.

## Highlights

• Readers with dyslexia do not have a selective deficit of global motion perception.
• The visual impairment in dyslexia is a difficulty processing temporal information.
• Global motion and form thresholds are significantly positively correlated.
• Gender and non-verbal IQ predict performance on random-dot global motion tasks.

## 1 Introduction

A predominant view is that human visual cortex is organised into two anatomically distinct and functionally independent processing streams or pathways, each specialised for encoding different types of visual information. The dorsal stream projects from primary visual cortex to the parietal lobes and is often referred to as the “where” pathway, as it is involved in tasks such as determining the global (overall) motion of objects, spatial cognition and visual motor planning. The ventral pathway projects from visual cortex to the temporal lobes and has been termed the “what” pathway, as it is involved in tasks such as global shape perception, visual memory and recognition of familiar objects/faces (Milner and Goodale, 1995, Ungerleider and Mishkin, 1982). Vulnerability of the dorsal stream has been suggested as a primary origin of impairment in individuals with developmental dyslexia, and a range of other neurodevelopmental disorders (e.g. Williams syndrome, autism spectrum disorder, developmental dyspraxia). Dorsal pathway vulnerability is claimed to manifest as a selective deficit in processing global motion relative to global form (Braddick, Atkinson, & Wattam-Bell, 2003). However the selectivity of this deficit is equivocal (Grinter, Maybery, & Badcock, 2010).

Several studies have used random-dot kinematograms (RDKs) to investigate the dorsal stream vulnerability hypothesis (see Benassi, Simonelli, Giovagnoli, & Bolzani, 2010 for review). These stimuli comprise a series of discrete images, each containing a pattern of individual local dots, that when presented in succession, create the perception of apparent motion. Some of the dots are constrained to move in a common direction (*signal* dots), whilst others move randomly (*noise* dots). By changing the relative proportion of signal and noise dots the coherence of the stimulus is varied. Motion coherence thresholds are defined as the minimum number of signals dots needed to detect or identify reliably the global motion direction (Britten et al., 1992, Newsome and Paré, 1988). To judge the overall direction of motion in a RDK local motion information has to be integrated (i.e. pooled, compared or combined) across two spatial dimensions and over time.

Cornelissen, Richardson, Mason, Fowler, and Stein (1995) were amongst the first to investigate the processing of global motion in poor readers classified as dyslexic. They administered a task originally devised by Wattam-Bell (1992). The stimuli comprised two RDKs. One of the patterns was segregated into three horizontal bands, whereas the other was spatially uniform. Signal dots in the former moved in opposite directions in adjacent bands. Those in the latter moved in a common direction. The participants’ task was to detect the segregated pattern. Consistent with the dorsal stream vulnerability hypothesis, poor readers’ coherence thresholds were significantly higher (1.3 times) than those of control readers. However, there was considerable heterogeneity in the performance of the two groups, a common finding in studies of developmental dyslexia (Amitay et al., 2002, Ramus et al., 2003, Roach et al., 2004, White et al., 2006), that recent research suggests might reflect genotypic variation (Cicchini et al., 2015, Gori et al., 2014).

The stimuli in the Cornelissen et al. (1995) study were spatially complex. To perform the task participants had to detect directional shearing between horizontal bands, rather than the direction of global motion *per se*. Thus one cannot determine whether poor readers have a difficulty processing visual motion in general or a difficulty detecting motion contrast. To address this issue, Raymond and Sorensen (1998) administered a simpler, conventional random-dot global motion task. A single RDK was presented on each trial, the participants had to judge the overall direction of the stimulus and motion coherence was varied. Poor readers coherence thresholds’ were significantly higher (1.8 times) than those of controls*.* However, there was no group difference when the RDKs consisted of only two images (i.e. the dots underwent a single displacement). These results imply that poor readers have a particular difficulty integrating local motion signals over extended trajectories, rather than a general difficulty with motion detection.

Talcott, Hansen, Assoku, and Stein (2000) sought to determine whether the perceptual deficit in poor readers reflects anomalous spatial or temporal integration. In two separate experiments, the mean dot density and exposure duration of random-dot stimuli, similar to those used by Raymond and Sorensen (1998) were manipulated. Results showed that overall poor readers’ coherence thresholds were significantly higher than those of normal readers in both experiments and there was no significant interaction between subject group and dot density nor subject group and duration, demonstrating that the spatiotemporal manipulations had similar effects regardless of reading ability. However, at the highest dot density tested (12.2 dots/deg^2^) the performance of readers with dyslexia approached that of the controls, suggesting a marginal improvement perhaps as a consequence of the greater motion energy present in the denser RDKs facilitating the poor readers. Talcott et al. speculated that greater motion energy might be expected to facilitate performance if motion sensors have a relatively low response gain, more inherent noise or sparser spatial sampling but no firm conclusions could be drawn.

An alternative hypothesis is that deficits on sensory tasks associated with poor reading and dyslexia are the result of impairments in external-noise exclusion (Sperling, Lu, Manis, & Seidenberg, 2005). Within this framework relatively poor performance on RDK tasks, in which coherence thresholds are used as a measure of sensitivity, is directly indicative of an underlying problem in segregating the signal dots from the noise dots. Although this noise-exclusion hypothesis has received support (e.g. Sperling, Lu, Manis, & Seidenberg, 2006) it fails to explain why some individuals with dyslexia often exhibit relatively normal performance on analogous static global form tasks that also contain high levels of visual noise. For example, Hansen, Stein, Orde, Winter, and Talcott (2001) administered two psychophysical tasks: a random-dot global motion task and a static global form task. The latter was devised by Atkinson et al. (1997) to investigate the processing of global form in individuals with Williams syndrome. It is assumed to provide a sensitive measure of ventral stream capability because it evokes a BOLD response in cortical areas that have been implicated in the processing of global form (Braddick, O’Brien, Wattam-Bell, Atkinson, & Turner, 2000). The stimuli in the task are similar to the random-dot patterns described above, except they comprise static line segments rather than dots. They can either be orientated coherently to form a concentric target or randomly. Poor readers’ coherence thresholds were significantly higher than those of controls on the random-dot global motion task but not the static global form task. This result is difficult to reconcile with a general noise-exclusion hypothesis but is consistent with the dorsal stream vulnerability hypothesis.

A related issue concerns the degree to which motion segmentation processes are normal in individuals with dyslexia. This is important because under natural viewing the visual system has to satisfy the competing requirements of integrating local motion signals that belong to a common surface or object but also segregating those arising from other objects in the world (e.g. Braddick, 1993). How the visual system achieves this delicate balance is still unknown but there is some evidence to suggest that motion segmentation mechanisms may also be impaired in poor readers. Hill and Raymond (2002) investigated this issue using transparent motion stimuli generated by constraining half of the dots in a RDK to move coherently in a horizontal direction (leftwards or rightwards) and others to move vertically (upwards or downwards). This created the perception of two segregated and transparent surfaces sliding across each other and the subjects’ task was to identify the two directions of motion present on each trial. The exposure duration of the stimulus was manipulated by changing the number of images comprising the motion sequence. A transparency threshold was calculated, which corresponded to the minimum exposure duration needed to achieve 75% correct performance. Results showed that poor readers’ transparency thresholds were over three times higher than those of controls in that they required an additional 339 ms to identify the two directions of simultaneous motion.

Recently it has been suggested that a deficit in the processing of global motion only occurs in a sub-group of individuals, which might explain why performance on random-dot tasks is heterogeneous (Amitay et al., 2002, Ramus et al., 2003, White et al., 2006). Approximately 10–17% of poor readers classified as dyslexic and 4% of controls have a deletion on intron 2 of the DCDC2 gene (Meng et al., 2005, Wilcke et al., 2009). Studies have shown that individuals with this genotypic deletion (hereafter referred to as DCDC2d) have altered white matter tracts in brain regions implicated in reading (e.g. Darki, Peyrard-Janvid, Matsson, Kere, & Klingberg, 2014). Interestingly, morphological changes have also been reported in extrastriate visual areas such as V5/MT (Morrone et al., 2011). Cicchini et al. (2015) administered a motion discrimination task to groups of poor readers with and without DCDC2d. The results showed that poor readers with the deletion had more profound impairments than those without DCDC2d. However, the latter performed significantly worse than controls, which suggests that factors other than genotypic variation are contributing to the inter-subject variability in coherence thresholds amongst poor readers.

An interesting question is whether a deficit in the processing of global motion is causal to developmental dyslexia. There is evidence to suggest that this might be the case (e.g. Gori, Seitz, Ronconi, Franceschini, & Facoetti, 2015) but these findings have not been replicated. Olulade, Napoliello, and Eden (2013) investigated whether motion-related activity in V5/MT differs between children with dyslexia and controls matched either in chronological- or reading-age. Significant differences would be expected for both types of comparison if causality were present (Goswami, 2014). Poor readers’ activity in V5/MT was significantly lower than that of chronological- but not reading-age matched controls. An eight-week phonological based intervention was then undertaken, which lead to significant improvements in poor readers’ scores on standardised measures of reading ability and increased neural activity in right V5/MT. Taken together, these findings imply that motion processing impairments are a consequence rather than the proximal cause of developmental dyslexia. Recent studies have suggested that the process of reading acquisition has an influence on the development of early visual areas (Carreiras et al., 2009, Dehaene et al., 2015, Szwed et al., 2012). The finer details of this hypothesis are still being worked out (see Grainger, Dufau, & Ziegler, 2016) but it could explain why some poor readers’ coherence thresholds are higher than those of good readers on random-dot global motion tasks.

**Fig. 1:**
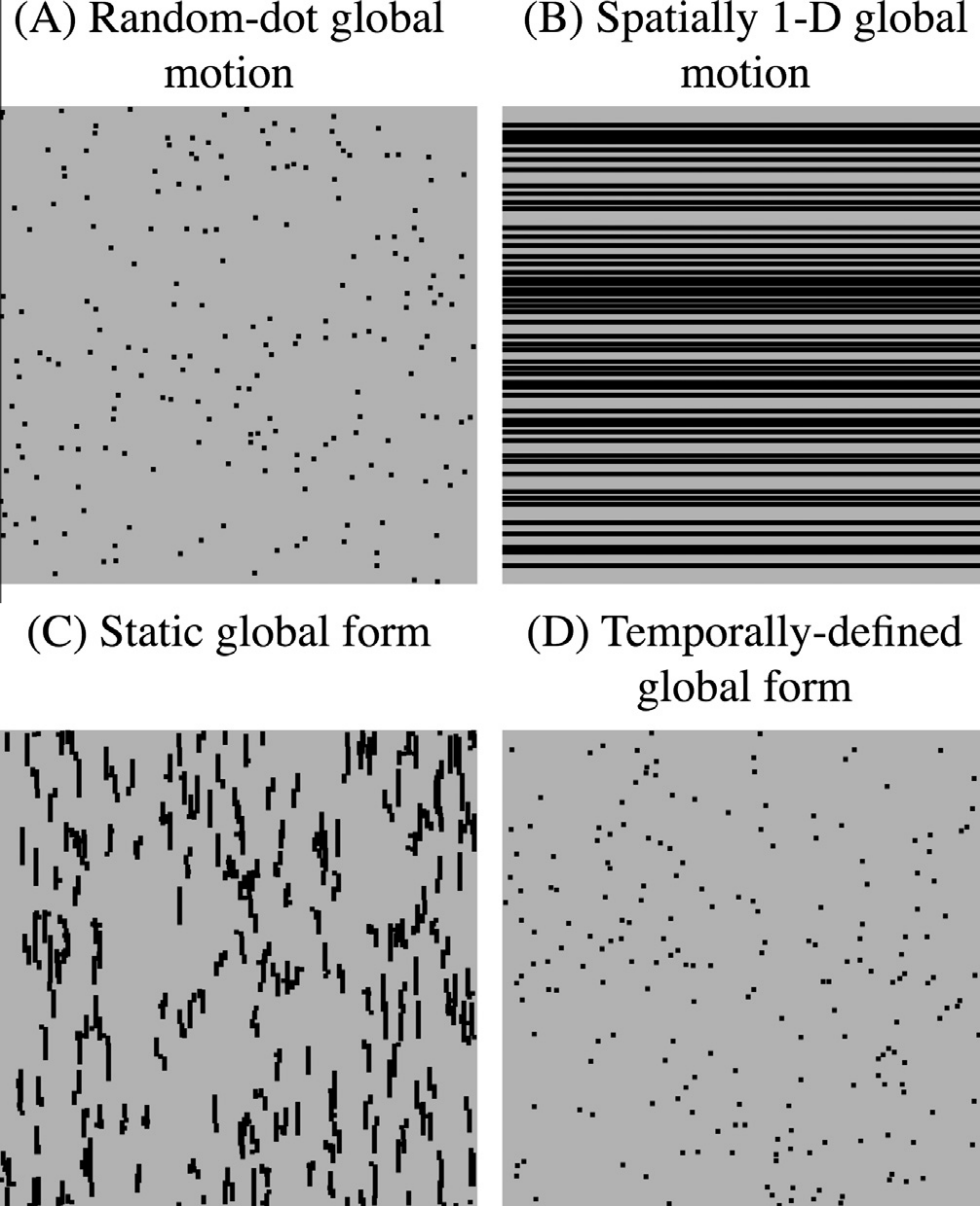
*Visual stimuli.* Schematic illustration of the visual stimuli used in (A) the random-dot global motion task, (B) the spatially 1-D global motion task, (C) the static global form task and (D) the temporally-defined global form task. Note that the temporally-defined global form task cannot be adequately depicted in this figure, as its apparent spatial structure arises from the asynchronous jittering of individual dots over time.

In summary, it appears that some individuals with dyslexia have a deficit on tasks involving global motion perception (Conlon et al., 2012, Conlon et al., 2013, Cornelissen et al., 1995, Everatt et al., 1999, Hansen et al., 2001, Hill and Raymond, 2002, Olulade et al., 2013, Pellicano and Gibson, 2008, Qian and Bi, 2014, Raymond and Sorensen, 1998, Ridder et al., 2001, Talcott et al., 2000, Talcott et al., 2003, Wilmer et al., 2004, Witton et al., 1998). However, the underlying nature of the perceptual deficit is unknown, a situation exacerbated by the fact that many studies have used arbitrary visual tasks. It may reflect a specific difficulty with motion detection, temporal processing or integrating local information across both dimensions of space and over time. Research has also failed to investigate factors that are associated with performance on random-dot global motion tasks such as gender and non-verbal IQ (Billino et al., 2008, Melnick et al., 2013, Snowdon and Kavanagh, 2006). To resolve these issues, we administered four, diagnostic, global motion and form tasks to a large sample of adult readers to characterise their perceptual abilities. These were: a random-dot global motion task, a spatially 1-D global motion task, a static global form task and a temporally-defined global form task (Fig. 1). Two sets of analyses were conducted. First, to investigate if general reading skills are associated with performance on each of the four visual tasks, a series of continuous regression analyses were conducted using a composite measure of reading ability with the whole sample of readers. Within these analyses we also investigated the influence of gender and non-verbal IQ on visual task performance. Second, to explore if performance across the four visual tasks differs across readers with dyslexia who had poor phonemic decoding skills and good readers, a series of between-group regression analyses were conducted. These groups were matched for non-verbal IQ. This enabled us to delineate performance on the visual tasks across individuals with developmental dyslexia compared to generally poor readers.

The four visual tasks administered were specifically designed to reveal the underlying nature of the perceptual deficit in readers with dyslexia. Specific predictions across these tasks are given in Table 1. If, as previously claimed, readers with dyslexia have a specific difficulty with motion detection, they would be expected to have higher coherence thresholds on both the random-dot global motion task and the spatially 1-D global motion task. If, on the other hand, the perceptual deficit in readers with dyslexia reflects a difficulty with temporal processing, they would be expected to have significantly higher coherence thresholds on the tasks requiring precise transmission of time-varying information, namely the random-dot global motion task, spatially 1-D global motion task and temporally-defined global form task. Finally, if readers with dyslexia have a difficulty confined to the most computationally-demanding tasks, requiring spatiotemporal integration of local information across multiple (>2) dimensions, they would be expected to have significantly impaired coherence thresholds on the random-dot global motion and temporally-defined global form tasks. Similar predictions could be made for individuals who are generally poor readers (using the composite measure of reading skill across the three reading tasks) if reading ability is shown to relate to performance on the visual tasks.

## 2 Materials and methods

### 2.1 Participants

A large sample of adults (*N* = 106; 64 female and 42 male) whose reading abilities ranged along a continuum was recruited to participate in the study, either via an undergraduate research participation scheme or Student Support Services at The University of Nottingham^1^. The latter was important in order to obtain sufficient participants with reading difficulties. The mean age of the participants was 22.02 years (SD = 62.47 months). Participants were required to have English as their first language and were excluded from the study if they had a neurodevelopment disorder other than developmental dyslexia (e.g. ADHD, developmental dyspraxia, autism spectrum disorder, amblyopia) or a history of ocular ill health. Research has found that individuals born pre-maturely typically have elevated coherence thresholds on random-dot global motion tasks (Taylor, Jakobson, Maurer, & Lewis, 2009), therefore participants were excluded if they were born less than thirty-two weeks gestation. All participants had normal or corrected-to-normal visual acuity. They gave informed consent to take part in this study according to the Declaration of Helsinki. The ethics committee at the School of Psychology, University of Nottingham, granted ethical approval for the study.

### 2.2 Psychometric tests

Each participant completed tests of non-verbal intelligence and reading ability. Non-verbal intelligence (IQ) was assessed using Raven’s Standard Progressive Matrices (SPM) (Raven, Court, & Raven, 1988). Three measures of reading ability were included that assessed different components of reading skill: (1) to assess whole-word lexical processing we administered the National Adult Reading Test (NART) (Nelson, 1991) which consists of 50 low-frequency irregular words; (2) to assess reading aloud of words that vary in frequency we gave the Test of Word Reading Efficiency (TOWRE) Sight Word Efficiency subtest (Torgesen, Wagner, & Rashotte, 1999); and (3) to assess sublexical decoding skills we administered the TOWRE Phonemic Decoding subtest. Participants are asked to read the words aloud and the number of errors is recorded. The TOWRE Sight Word Efficiency subtest and the TOWRE Phonemic decoding subtest are both speeded tests whereas the NART is self-paced. The TOWRE Sight Word Efficiency subtest measures speeded reading of 104 regular words, which vary in frequency. The TOWRE Phonemic decoding subtest measures speeded reading of 63 nonsense words varying in complexity. In both of these speeded tests participants are given 45 s to read as many words as possible. For each of the three reading tasks the dependent variable was the number of words read correctly.

### 2.3 Visual stimuli

Four visual tasks were generated that differentiated global motion from global form processing and enabled specific predictions to be made (see Fig. 1). The stimuli in each of these tasks were generated using a *Macintosh G5* computer and custom software written in the “C” programming language. The tasks were administered in a darkened vision laboratory and displayed on an *Intergraph Interview 24hd96* monitor (frame refresh rate of 75 Hz), which was carefully gamma-corrected using a photometer and look-up-tables. As an additional precaution, psychophysical procedures were used to check the adequacy of the gamma-correction (Ledgeway and Smith, 1994, Nishida et al., 1997).

Stimuli were viewed binocularly at a distance of 114 cm and were presented within the confines of a square display window in the centre of the monitor, subtending 12° × 12°. Each stimulus was composed of an ensemble of “black” elements (0.01 cd/m^2^), either dots (diameter 0.12°) or elongated bars (0.12° × 12°), presented against a uniform “grey” (34 cd/m^2^) background. These elements were either static or could be made to move or flicker depending on the nature of the visual task employed. The total stimulus duration in each case was 0.43 s. Specific details related to stimulus generation in each of the four visual tasks are given below.

#### 2.3.1 Random-dot global motion task

Stimuli in the random-dot global motion task (Fig. 1A) were conventional RDKs. They consisted of eight images, each containing 200 dots that were presented consecutively at a rate of 18.75 Hz to create the perception of apparent motion. Each individual dot was displaced by 0.12° on each update, resulting in a drift speed of 2.26°/s. The “strength” or coherence of the stimulus could be varied between 0 and 100% by constraining some of the dots to move in a common direction (*signal* dots) and the remainder to move randomly (*noise* dots). Whether an individual dot was assigned to be signal or noise was randomised on every displacement, so the direction in which that dot moved was limited in time. The subjects’ task was to judge the global (overall) direction of the RDK, which was chosen to be upwards or downwards on each trial with equal probability. This task required the integration of local information across two spatial dimensions and over time (*x*, *y*, & *t*).

#### 2.3.2 Spatially one-dimensional (1-D) global motion task

Stimuli in the spatially 1-D global motion task (Fig. 1B) were directly analogous to the random-dot global motion patterns previously described, except they comprised 50 horizontal bars, rather than dots. The coherence of the stimulus could be varied between 0 and 100% by constraining some the bars to move in a common direction (*signal* bars) and others to move randomly (*noise* bars). As in the random-dot global motion task, the speed of the bars was identical (2.26°/s), regardless of whether they were assigned to be signal or noise. Again, the subject’s task was the judge the global direction of the stimulus, which was chosen to be upwards or downwards on each trial with equal probability. This task required the integration of local information across one dimension of space and over time (*y* & *t*).

#### 2.3.3 Static global form task

Stimuli for the static global form task (Fig. 1C) were generated using a full 8-frame random-dot global motion sequence. The individual frames were then spatially superimposed to create a static image (Simmers, Ledgeway, & Hess, 2005). Some of the dots in the stimulus (*signal* dots) formed localised streaks, orientated along a common axis, whilst others (*noise* dots) formed random groupings. By changing the relative proportions of signal to noise dots in the image the coherence of the stimulus could be varied between 0 and 100%. The subjects’ task was the judge the overall orientation of the stimulus, which was chosen to be vertical or horizontal on each trial with equal probability. This required local information to be integrated across two dimensions of space (*x* & *y*).

#### 2.3.4 Temporally-defined global form task

Stimuli in the temporally-defined global form task (Fig. 1D) consisted of 200 dots that could be randomly replotted asynchronously at a rate of 18.75 Hz. Half the dots (population 1) were spatially jittered, whilst the other half (population 2) remained the static. The converse then occurred, and so on throughout the presentation. An orientated boundary was created by constraining more of population 1 to fall in one half of the display and population 2 in the opposing part. The coherence of the temporal information, giving rise to the perceptual boundary, could be varied between 0 and 100%. The subjects’ task was to judge the overall orientation of the perceptual boundary, which was chosen to be vertical or horizontal on each trial with equal probability. This task required local information to be integrated or compared across two spatial dimensions and over time (*x*, *y*, & *t*).

### 2.4 Procedure

Participants were first given the three different measures of reading ability, after which the four visual tasks were administered. Coherence thresholds were obtained for each of the visual tasks using a single-interval, forced-choice procedure and a 3-down, 1-up adaptive staircase tracking the 79.3% correct performance level. The staircase’s initial step size was equal to the total number of elements in the display and this decreased by half after each reversal. The staircase terminated when the number of reversals with a step size equal to one element (either a dot or a bar depending on the visual task employed) reached six. The arithmetic mean of the last six reversals was the threshold estimated from that staircase. The reported coherence threshold for each subject corresponds to the mean of at least four staircases and the order of testing was randomised across the four visual tasks. Finally, the measure of non-verbal intelligence was given.

### 2.5 Statistical analyses

The whole-sample and between-group regression analyses are outlined below. In both types of analyses, raw coherence thresholds on the visual tasks were used as dependent variables and an alpha-level of 0.05 was used to determine significance.

#### 2.5.1 Regression analyses: Whole-sample

These analyses were conducted to explore how general reading performance relates to performance on each of the four visual tasks, so a composite measure of reading ability was needed. First, to generate the composite measure of reading ability for the entire sample (*N* = 106), scores from each of the three reading tests were z-transformed, to allow direct comparisons to be made. Bivariate correlations (Pearson’s product-moment correlation coefficient) were then performed to investigate the relationships between the individual measures of reading ability.

Next, to investigate if general reading ability (using the composite score) is associated with performance on each of the four visual tasks, a regression model was built for each task with coherence threshold as the dependent variable. As previous research has found that gender and non-verbal IQ are associated with performance on tasks requiring motion processing (Billino et al., 2008, Hutchinson et al., 2012, Melnick et al., 2013, Snowdon and Kavanagh, 2006), these were entered as control variables at step 1. Scores for the composite reading measure of Reading Skill were introduced at step 2. We evaluated the R^2^ change at step 2 to determine if reading ability explained any additional variance after controlling for the effects of Gender and Non-Verbal IQ. A variation of Cohen’s *f*^2^ was used to calculate local effect size with 0.02 considered a small, 0.15 a medium and 0.35 a large effect, respectively (Cohen, 1988, Selya et al., 2012).

#### 2.5.2 Regression analyses: Between-group

To investigate if the performance of individuals that have poor phonemic decoding skills, consistent with the dyslexic profile (Snowling, 2000), differs from that of good readers across the four visual tasks, a series of between-group regression analyses were conducted. Evaluation of the individual measures of reading ability revealed forty-three participants (40.57% of the entire sample) had standard scores less than or equal to 85 (at or below the 15th percentile) on the TOWRE Phonemic Decoding subtest, which falls into the conventional range for identifying individuals with developmental dyslexia (Heath et al., 2006, Pugh et al., 2014). Performance of this group of readers with dyslexia was compared to that of relatively good readers who did not exhibit a phonological deficit. To identify the group of good readers standard scores on the TOWRE Phonemic Decoding subtest were ranked and the top forty-three individuals were selected (range of standard scores = 93–120). This ensured a balanced design in which all of the good readers’ scores were either within, or better, than the normal range (±1SD) on the TOWRE Phonemic Decoding subtest. The group of readers with dyslexia (identified by poor phonemic decoding skills) also had significantly lower scores than the group of good readers on the NART and TOWRE Sight Word Efficiency subtest, as reported in Table 2. Importantly, there was no significant group difference on the SPM measure of non-verbal IQ (Table 2), hence any differences in the performance of the dyslexia group compared to the good readers on the four visual tasks cannot be attributed to differences in non-verbal intelligence.

A series of regression analyses were then conducted to compare the performance of readers with dyslexia and good readers across the four visual tasks. For each task, a model was built with coherence threshold as the dependent variable. As above, Gender and Non-Verbal IQ were entered at step 1 as control variables then Reading Group (Good = 0; Dyslexia = 1) was introduced at step 2. We studied the R^2^ change to determine if Reading Group explained any additional variance after controlling for the effects of Gender and Non-Verbal IQ. As above, Cohen’s *f*^2^ was used to calculate local effect size.

#### 2.5.3 Independence of visual tasks

Finally, we wanted to investigate the proposed independence of the dorsal and ventral processing streams, as measured by tasks of global motion and global form perception (Milner and Goodale, 1995, Ungerleider and Mishkin, 1982). To explore relationships between the four psychophysical tasks across the entire sample (*N* = 106) raw coherence thresholds were z-transformed for each task, and then bivariate correlations (Pearson’s product-moment correlation coefficient) were conducted.

## 3 Results

Results from the two sets of regression analyses are given below.

### 3.1 Regression analyses: Whole-sample

**Fig. 2:**
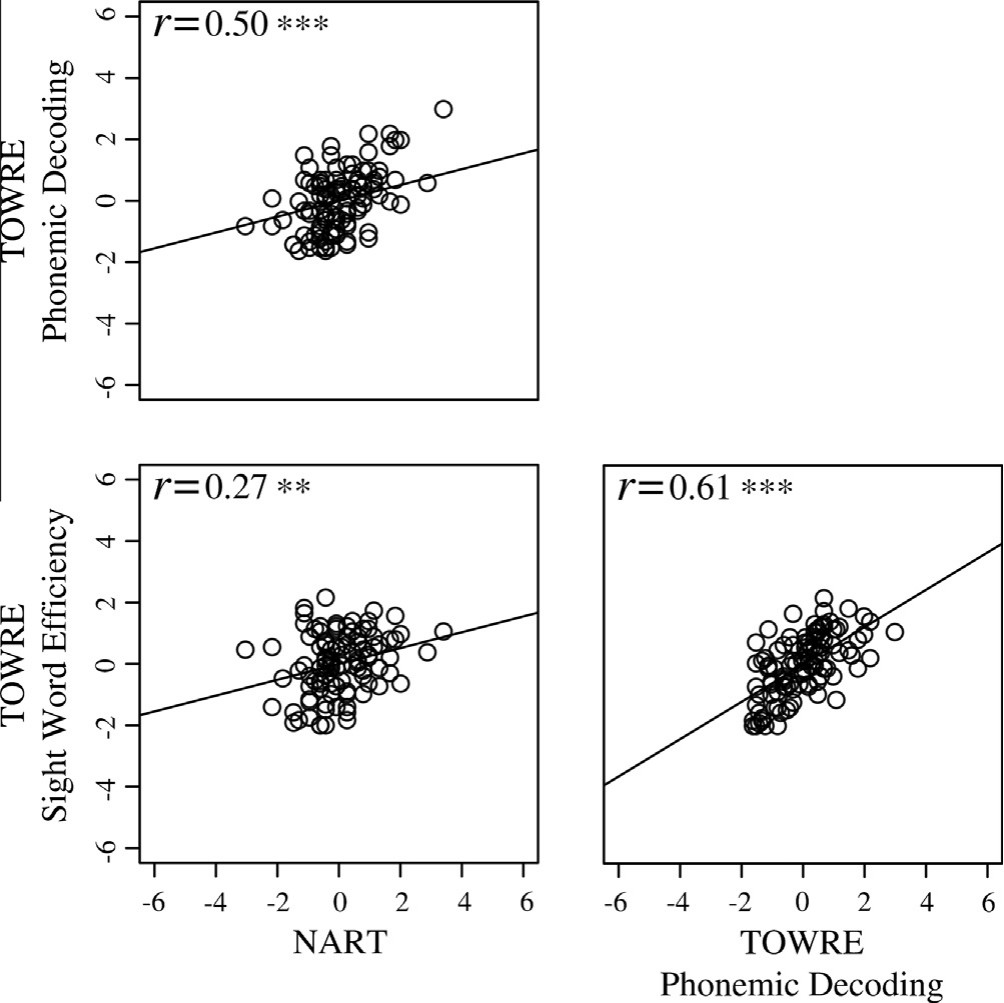
*Bivariate correlations: individual measures of reading ability.* Scatterplots showing the relationships between scores for the individual measures of reading ability in the entire sample (*N* = 106). Positive and negative z-scores indicate scores greater than and less than the mean of the sample, respectively. ^∗^*p* < 0.05, ^∗∗^*p* < 0.01, ^∗∗∗^*p* < 0.001. NART = National Adult Reading Test; TOWRE = Test of Word Reading Efficiency.

Correlations between scores from the individual measures of reading ability were weak to moderate (*r* = 0.27–0.61, see Fig. 2) so principal component analysis (PCA) was conducted to calculate the composite measure of reading skill. Raw scores for the three reading tests were entered into the analysis, which was based on the correlation matrix. This implicitly accomplishes the transformation from raw scores to standard scores. Results showed a single principal component that accounted for 64% of the total variance amongst the three measures of reading ability (eigenvalue 1 = 1.93; eigenvalue 2 = 0.74; eigenvalue 3 = 0.33). Loadings for the NART and the TOWRE Sight Word Efficiency subtest were within the same range (0.71 and 0.79, respectively) but the TOWRE Phonemic Decoding subtest contributed more to the construct (loading = 0.90). Principal component scores were thus extracted for each participant in the entire sample and entered into the whole-sample regression analyses. Table 3 reports the raw coherence thresholds for the four visual tasks across the entire sample. Regression analysis results for each of the visual tasks are reported in Table 4 and described in the sections below.

#### 3.1.1 Random-dot global motion

In the regression model for the random-dot global motion task, the control variables explained 16% of the variance, *F*_2, 103_ = 9.79, *p* < 0.001. Gender was associated with performance on the task. Females’ coherence thresholds were significantly higher than those of males. In addition, Non-Verbal IQ was a significant predictor of performance. Individuals with a lower IQ had higher coherence thresholds on the random-dot global motion task. At step two, the R^2^ change was significant, *F*_1, 102_ = 7.80, *p* *<* 0.01. General Reading Skill was negatively associated with performance on the task. It explained an additional 6% of the variance after controlling for the effects of Gender and Non-Verbal IQ. Coherence thresholds were elevated in those who were generally poor at reading i.e. had lower scores on the composite measure of reading skill.

#### 3.1.2 Spatially 1-D global motion

The control variables explained 8% of the variance in the model for the spatially 1-D global motion task, *F*_2, 103_ = 4.33, *p* = 0.02. There was no effect of Gender but Non-Verbal IQ was a significant predictor of performance. Individuals with lower IQ had higher coherence thresholds on the spatially 1-D global motion task. The R^2^ change at step two was significant, *F*_1, 102_ = 5.15, *p* = 0*.*03. General Reading Skill was negatively associated with performance on the task. It explained an additional 4% of the variance after controlling for the effects of Gender and Non-Verbal IQ. Coherence thresholds were higher in those who had lower composite scores for reading.

#### 3.1.3 Static global form

In the model for the static global form task, the control variables did not explain a significant amount of the variance, *F*_2, 103_ = 0.28, *p* = 0.76. Furthermore, the R^2^ change at step two did not reach statistical significance, *F*_1, 102_ = 0.20, *p* = 0*.*65. Reading Skill was not associated with performance on the static global form task.

#### 3.1.4 Temporally-defined global form

Gender and Non-Verbal IQ did not explain a significant amount of variance in the model for the temporally-defined global form task, *F*_2, 103_ = 1.60*, p* = 0.21. However, the R^2^ change at step two was significant, *F*_1, 102_ = 11.16, *p* *<* 0*.*01. General Reading Skill was negatively associated with performance on the task. It explained an additional 10% of the variance after controlling for the effects of Gender and Non-Verbal IQ. Coherence thresholds were elevated in those who were generally poor at reading compared to those with higher reading scores.

### 3.2 Regression analyses: Between-group

Results from the series of between-group regression analyses revealed significant group differences for some of the visual tasks. Raw coherence thresholds for the group of readers with dyslexia and relatively good readers are reported in Table 5. For each task, results from the between-group regression analyses are reported in Table 6 and described in the sections below.

#### 3.2.1 Random-dot global motion

In the regression model for the random-dot global motion task, the control variables explained 19% of the variance, *F*_2, 83_ = 9.89, *p* *<* 0.001. Gender was associated with performance on the task. Females’ coherence thresholds were significantly higher than those of males. In addition, Non-Verbal IQ was a significant predictor of performance. Individuals with a lower IQ had higher coherence thresholds on the random-dot global motion task. At step two, the R^2^ change was significant, *F*_1, 82_ = 8.25, *p* *<* 0.01. Reading Group was associated with performance on the task. It explained an additional 8% of the variance after controlling for the effects of Gender and Non-Verbal IQ. Coherence thresholds were significantly higher in readers with dyslexia who had poor phonemic decoding skills.

#### 3.2.2 Spatially 1-D global motion

The control variables explained 6% of the variance in the model for the spatially 1-D global motion task, *F*_2, 83_ = 2.55, *p* *=* 0.08. There was no significant effect of Gender but Non-Verbal IQ was negatively associated with performance. Individuals with a lower IQ had higher coherence thresholds on the spatially 1-D global motion task. The R^2^ change at step two approached but did not reach statistical significance, *F*_1, 82_ = 3.20, *p* *=* 0.08. Coherence thresholds did not differ between the two reader groups. However, there was a non-significant trend.

#### 3.2.3 Static global form

Gender and Non-Verbal IQ did not explain a significant amount of variance in the model for static global form task, *F*_2, 83_ = 0.11, *p* *=* 0.90. Moreover, the R^2^ change at step two failed to reach statistical significance, *F*_1, 82_ *=* 0.10, *p* *=* 0.75. Reading Group was not associated with performance on the static global form task. Coherence thresholds did not differ significantly between the two groups.

#### 3.2.4 Temporally-defined global form

The control variables did not explain a significant amount of variance in the model for the temporally-defined global form task, *F*_2, 83_ = 1.49, *p* *=* 0.23. However, the R^2^ change at step two was significant, *F*_1, 82_ *=* 6.02, *p* *=* 0*.*02. Reading Group was associated with performance on the task. It explained an additional 7% of the variance after controlling for the effects of Gender and Non-Verbal IQ. Coherence thresholds were significantly higher in the group of readers with dyslexia who had poor phonemic decoding skills.

### 3.3 Independence of visual tasks

**Fig. 3:**
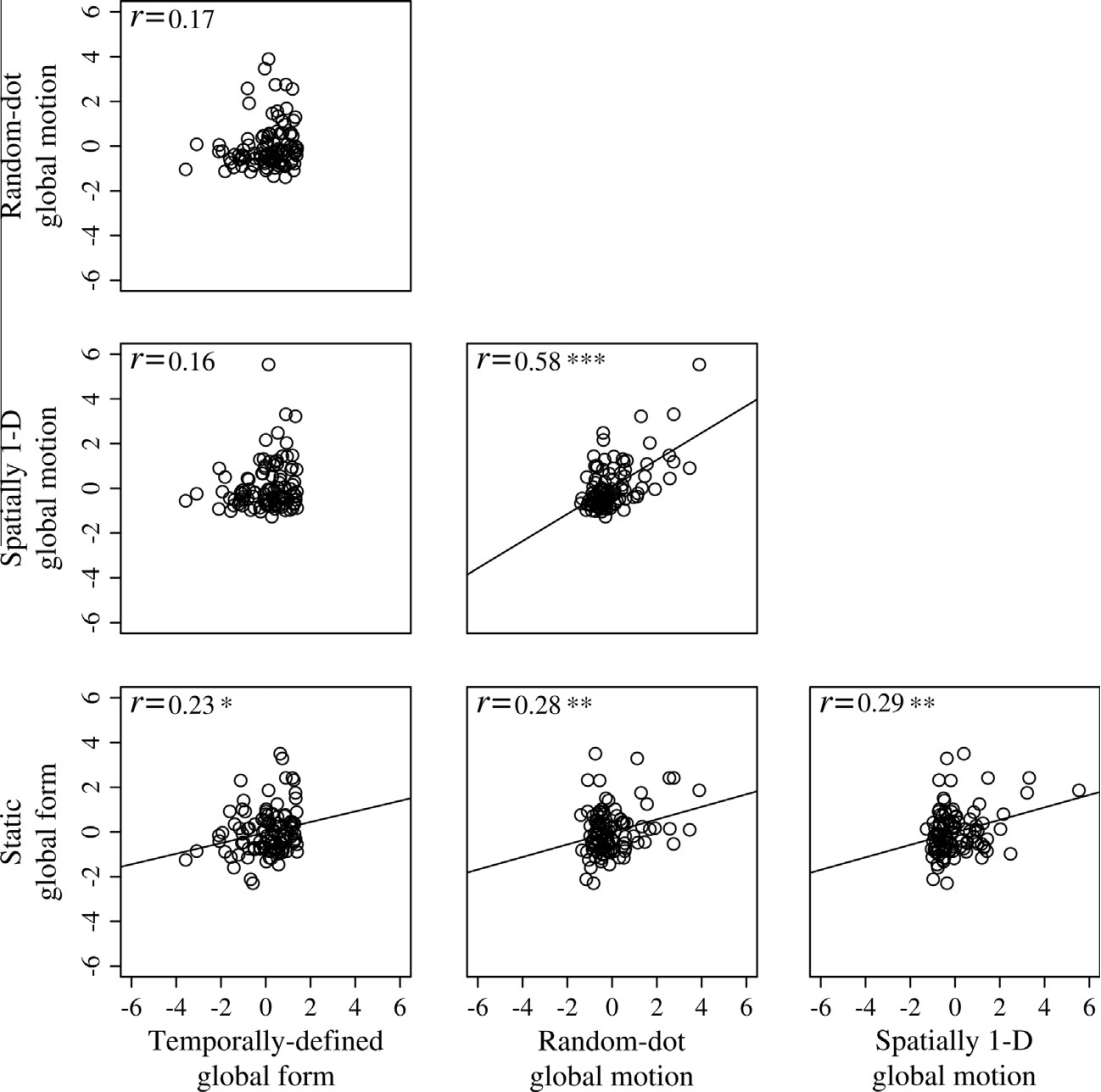
*Bivariate correlations: visual tasks.* Scatterplots showing the relationships between coherence thresholds for the visual tasks in the entire sample (*N* = 106). Positive and negative z-scores indicate coherence thresholds greater than and less than the mean of the sample, respectively. ^∗^ p < 0.05, ^∗∗^ p < 0.01, ^∗∗∗^ p < 0.001.

Scatterplots illustrating performance of the entire sample across the four visual tasks are given in Fig. 3. As expected, a strong and significant correlation was found between thresholds across the two global motion tasks and the two global form tasks (random-dot global motion task and spatially 1-D global motion task, *r*_106_ = 0.58, *p* < 0.001; static global form task and temporally-defined global form task, *r*_106_ = 0.23, *p* = 0.02). However, significant and positive correlations were also found across tasks measuring the processing of global motion and global form (random-dot global motion task and static global form task, *r*_106_ *=* 0.28, *p* < 0.01; spatially 1-D global motion task and static global form task, *r*_106_ = 0.29, *p* < 0.01). In contrast, no significant correlation was found between the spatially 1-D global motion task and the temporally-defined global form task, *r*_106_ = 0.16, *p* = 0.10 or the random-dot global motion task and the temporally-defined global form task, *r*_106_ = 0.17, *p* = 0.09.

## 4 Discussion

The present study explored why readers with dyslexia typically exhibit relatively impaired performance on tasks involving the perception of global motion but not those involving the perception of static global form. To investigate this issue, we tested the perceptual abilities of a large undifferentiated sample of readers using a novel stimulus paradigm that allowed us to establish the underlying nature of the reported deficit in individuals with, and without, dyslexia. Our tasks enable us to differentiate between explanations based upon difficulties with motion detection, temporal processing or spatiotemporal integration as the number of stimulus dimensions increases.

A similar pattern of results was found across the whole-sample analyses, involving the entire sample, and the between-group analyses, comparing performance of readers with dyslexia who had poor phonemic decoding skills and relatively good readers. Consistent with previous studies, we found that the coherence thresholds of readers with dyslexia were significantly higher than those of relatively good readers on the random-dot global motion task but not the static global form task (Hansen et al., 2001). The same pattern was found for generally poor readers. In addition, with the novel task of spatially 1-D global motion, both generally poor readers and individuals with dyslexia showed elevated coherence thresholds compared to good readers although this difference did not reach significance in the between-group analyses (*p* = 0.08, 2-tailed). However, the amount of variance explained after controlling for the effects of Gender and Non-Verbal IQ was similar across analyses (whole-sample analyses = 4% of total variance; between-group analyses = 3% of total variance), consistent with a modest deficit in the processing of 1-D global motion. We also found in both analyses that reading ability/group significantly predicted coherence thresholds on the temporally-defined global form task, as both readers with dyslexia and generally poor readers showed elevated thresholds on this task compared to relatively good readers. This unique finding is difficult to reconcile with the dorsal stream vulnerability hypothesis (Braddick et al., 2003).

The consistent pattern of results found across the four visual tasks for readers with dyslexia, who had poor phonemic decoding skills, and generally poor readers suggest that visual difficulties do not differentiate these two groups of poor readers. This is important to demonstrate as some argue that dyslexia best represents the lower-end of a normal distribution of reading ability, whilst others suggest it is a distinct type of reading difficulty (Fletcher, 2009, Shaywitz et al., 1992, Siegel, 2006). It is possible that readers with dyslexia differ from generally poor readers on other tasks, but we have clearly shown that on tasks of global motion and global form processing they perform similarly to generally poor readers.

Taken together, the results of the whole-sample and between-group analyses demonstrate that the underlying nature of the visual deficit in readers with dyslexia and generally poor readers reflects a difficulty processing temporal, rather than motion, information *per se*. An interesting question is whether this impairment generalises to other sensory domains. Recently, it has been suggested that auditory temporal sampling is impaired in poor readers (Goswami, 2011). Within this framework, spoken words are encoded by phase-locking of brain activity in different frequency bands. Low-frequency gamma oscillations (25–45 Hz) are dominant in the left hemisphere and have been implicated in the analyses of phonemes, whereas delta-theta rhythms (1–7 Hz) are lateralised to the right hemisphere and are thought to play a major role in the processing of syllabic and prosodic cues (Poeppel, 2003). There is debate as to whether slow or fast sampling is abnormal in poor readers but recent studies support the view that auditory entrainment in the gamma frequency band is impaired (Lehongre et al., 2013, Lehongre et al., 2011). This is thought to manifest as a deficit with the temporal segmentation of phonemic units in the speech stream (Giraud & Ramus, 2013).

Furthermore, our results also suggest that the visual deficit is exacerbated when local visual cues have to be integrated across multiple (>2) dimensions. Impairment was most marked on the random-dot global motion task and the temporally-defined global form task, as indicated by the effect sizes in Table 4, Table 6. Both of these tasks required integration of local visual cues across two dimensions of space as well as over time. If this explanation is valid then generally poor readers and individuals with dyslexia should also exhibit deficits on a range of other visual tasks. For example, accurately encoding the global motion of an object defined purely by stereoscopic (cyclopean) depth cues requires combination of visual information across four dimensions (*x*, *y*, *z*, & *t*) and may be extremely challenging for the least skilled readers. Future research aimed at testing this and related predictions should help to refine the contribution of task complexity to the profile of visual impairment in dyslexia.

Our results also cast further doubt on the noise-exclusion hypothesis of dyslexia, since a difficulty in segregating signal from noise elements would be expected to impair performance on all four visual tasks but this was not the case. This suggests that noise exclusion (Sperling et al., 2005, Sperling et al., 2006) in itself is not the proximal cause of the perceptual deficit shown in generally poor readers and individuals with dyslexia. However, it may be possible to reconcile this theory with the present results if we assume that these individuals exhibit some difficulties with external-noise exclusion but only when high levels of noise are present in tasks that require integration of visual information over time. A recent study has shown that readers with dyslexia have elevated levels of choline and glutamate in visual cortex, leading to hyperexcitability and increased susceptibility to noise (Che et al., 2014, Pugh et al., 2014). Consequently, it would be interesting to investigate if coherence thresholds on the spatially 1-D global motion task, the random-dot global motion task, and the temporally-defined global form task are associated with neurometabolic concentration in visual cortex.

It is interesting to note that after controlling for the effects of Gender and Non-Verbal IQ, Reading skill explained more of the variance (10%) in performance on the temporally-defined global form task than any of the other visual tasks. Unlike the other three visual tasks the temporally-defined form task requires some degree of segmentation, as well as integration, of local cues. That is, to identify the global orientation of the perceptual boundary visual information provided by temporally asynchronous jitter cues must be integrated within each half of the display but also segmented from those in the opposing half of the image. That reading ability was the strongest predictor of performance on this particular task is consistent with previous studies that have investigated motion segmentation in poor readers (Hill & Raymond, 2002).

Our results also showed that Non-Verbal IQ was negatively associated with coherence thresholds on the spatially 1-D global motion task and the random-dot global motion task in both the whole-sample and between-group analyses. Previous research has reported a link between intelligence and motion processing (Melnick et al., 2013). The differential performance of individuals with relatively low and high IQs might reflect differences in spatial suppression; an inhibitory process that reduces the response of some neurons in area MT/V5 to large background-like stimuli (Tadin, Lappin, Gilroy, & Blake, 2003). It has been suggested that individuals with a high IQ have an enhanced ability to suppress ecologically less relevant information in the visual field. In contrast, intelligence was not associated with thresholds on the static global form task nor the temporally-defined global form task. Further research needs to establish why non-verbal IQ appears to be associated with performance on global motion tasks but not those involving analogous global form.

Gender was also a significant predictor of thresholds on the random-dot global motion task. Females’ coherence thresholds were significantly higher (1.3 times) than those of males, consistent with some previous research (Billino et al., 2008, Snowdon and Kavanagh, 2006). The fact that gender was not significantly associated with performance on the temporally-defined global form task suggests that some females have a specific difficulty on random-dot global motion tasks, which is distinct from the temporal processing impairment exhibited by generally poor readers and individuals with dyslexia. Although speculative, this gender effect might reflect differences in inter-hemispheric asymmetry. For example, extrastriate motion area MT/V5 in the right hemisphere of the male is reported to have a significantly larger volume than the corresponding region in the female cortex (Amunts et al., 2007, de Lacoste et al., 1991, Kovalev et al., 2003). It has been suggested that this provides additional neural resources or “space” for the processing of computationally-demanding visual stimuli. To some extent, the results of the current study are consistent with this hypothesis, given that gender was not associated with coherence thresholds for the simpler spatially 1-D global motion task. Further research is needed to determine why gender does not significantly predict coherence thresholds for global form tasks. A highly tentative possibility is that the parts of the brain involved in the processing of global form are not characterised by the same left-right asymmetry that is observed in area MT/V5 of the male. Regardless of the underlying mechanism of the gender effect, that females have typically higher thresholds than males for random-dot global motion, could explain why some studies have failed to find differences between reading groups on this task (Amitay et al., 2002, White et al., 2006). For example, very marked gender imbalances between participant groups (i.e. more females than males in the control group and vice versa for the group of readers with dyslexia) could potentially mask differences in performance driven by reading ability. Thus future studies need to control for gender when performing between-group analysis.

On a related note, the results of the between-group analyses showed that there was considerable inter-subject variability in coherence thresholds amongst the group of readers with dyslexia even after controlling for the effects of Gender and Non-Verbal IQ. This is consistent with previous studies exploring sensory theories of developmental dyslexia (Amitay et al., 2002, Ramus et al., 2003, Roach et al., 2004). It was especially marked for the two global motion tasks, as indicated by the relatively large standard deviations in Table 5. A potential explanation for this is that visual deficits only occur in a sub-group of readers with dyslexia. Some have argued that this might reflect genotypic variation (e.g. Cicchini et al., 2015) but further research is needed to establish this. Interestingly, the intra-subject variability (i.e. variability in each individual’s thresholds measured across different staircases) was only slightly (and not significantly) higher in readers with dyslexia (average SD = 9.08%) than in good readers (average SD = 7.41%), suggesting that an individual’s reading ability does not greatly affect the reliability of their performance across trials on the visual tasks.

Finally, our results also have a direct bearing on the proposed independence of the dorsal and ventral processing streams and the types of information that each pathway encodes. Consistent with the two-streams hypothesis, across the entire sample we found a significant, positive correlation between coherence thresholds for the two global motion tasks and also for the two global form tasks. However, the results that are difficult to reconcile with the dorsal-ventral dichotomy are the significant correlations between thresholds for the static global form task and the two global motion tasks. This latter result is important because it is indicative of some degree of cross-talk between the dorsal and ventral streams, or the existence of a later common processing stage that serves to provide a unified representation of an object’s global properties. There is mounting evidence from other psychophysical studies that the processing of global motion and global form is not strictly independent (see Mather, Pavan, Marotti, Campana, & Casco, 2013 for a review). For example, Ross, Badcock, and Hayes (2000) showed that a series of temporally uncorrelated Glass patterns, containing global oriented structure created by pairs of dots related by a rotational shift, induced precepts of global rotational motion consistent with the spatial information. Clearly, this has implications for future studies and for the interpretation of previous findings.

## 5 Conclusions

The results of this study indicate that the visual deficit in both generally poor readers and individuals with dyslexia is better characterised as a difficulty processing temporal, rather than motion information *per se*. Furthermore, our results suggest that the processing of global motion and global form are not strictly independent in the human visual system as we found significant, positive correlations between thresholds for the static global form task and the two global motion tasks. Thus, the use of global motion and global form tasks in vision experiments cannot be guaranteed to readily dissociate activity in the dorsal and ventral processing streams.

## Figures and Tables

**Table 1:** Predicting performance on the visual tasks.

Source of difficulty	Impaired	Normal
Motion processing	Random-dot global motion	Static global form
	Spatially 1-D global motion	Temporally-defined global form

Temporal processing	Random-dot global motion	Static global form
	Spatially 1-D global motion	
	Temporally-defined global form	

Multi-dimensional integration (>2 dimensions)	Random-dot global motion	Spatially 1-D global motion
	Temporally-defined global form	Static global form

Impaired = Readers with dyslexia expected to have significantly higher coherence thresholds than good readers; Normal = no significant difference expected between good readers and readers with dyslexia.

**Table 2:** Group psychometric statistics.

	Dyslexia (*N* = 43)			Good (*N* = 43)			*t*_84_
	*M*	*Median*	*SD*	*M*	*Median*	*SD*	
NART (raw score/50)	23.72	24.00	6.03	29.63	29.00	4.32	5.22⁎⁎⁎
TOWRE Sight Word Efficiency	78.16	77.00	6.24	92.53	90.00	12.06	6.94⁎⁎⁎
TOWRE Phonemic Decoding	80.72	83.00	4.91	104.33	103.00	9.61	14.34⁎⁎⁎
SPM (raw score/60)	50.02	51.00	5.63	50.79	51.00	3.91	0.73

Standard scores (*M* = 100, *SD* = 15) are shown unless otherwise stated. NART = National Adult Reading Test; TOWRE = Test of Word Reading Efficiency; SPM = Raven’s Standard Progressive Matrices. ^∗^p < 0.05. ^∗∗^p < 0.01.

⁎⁎⁎ *p* < 0.001.

**Table 3:** Coherence threshold (%) statistics for the entire sample (*N* = 106).

	Male (*N* = 42)			Female (*N* = 64)		
	*M*	*Median*	*SD*	*M*	*Median*	*SD*
Random-dot global motion	16.80	15.64	6.68	21.89	18.47	10.78
Spatially 1-D global motion	18.74	13.97	11.32	18.15	15.53	11.67
Static global form	14.87	14.04	4.31	14.90	14.54	3.81
Temporally-defined global form	91.47	91.88	6.10	91.96	92.92	5.45

**Table 4:** *Regression analyses: Whole-sample.* A model was run for each visual task with threshold as the dependent variable. The control variables (i.e. Gender and Non-Verbal IQ) were entered into the models at step one. Reading Skill (derived from the PCA) was introduced at step two. The performance of the entire sample was considered. Statistically significant results are shown in bold font.

Task	*N*	Step	*R^2^*	Δ*R^2^*	*B*	SE B	β	Cohen’s *f*^2^
Random-dot global motion	106	Step 1	**0.16**⁎⁎⁎					
		Gender			**4.48**	**1.79**	**0.23**⁎	**0.06**
		SPM			**−0.63**	**0.19**	**−0.31**⁎⁎	**0.11**
		Step 2	**0.22**⁎⁎⁎	**0.06**⁎⁎				
		Gender			**4.79**	**1.73**	**0.24**⁎⁎	
		SPM			**−0.56**	**0.18**	**−0.27**⁎⁎	
		Reading skill			**−2.39**	**0.85**	**−0.25**⁎⁎	**0.08**

Spatially 1-D global motion	106	Step 1	**0.08**⁎					
		Gender			−1.25	2.22	−0.05	
		SPM			**−0.68**	**0.23**	**−0.28**⁎⁎	**0.09**
		Step 2	**0.12**⁎⁎	**0.04**⁎				
		Gender			−0.94	2.18	−0.04	
		SPM			**−0.61**	**0.23**	**−0.25**⁎⁎	
		Reading skill			**−2.44**	**1.08**	**−0.21**⁎	**0.05**

Static global form	106	Step 1	0.01					
		Gender			−0.03	0.80	−0.00	
		SPM			−0.06	0.08	−0.07	
		Step 2	0.01	0.00				
		Gender			−0.01	0.81	−0.00	
		SPM			−0.06	0.09	−0.07	
		Reading skill			−0.18	0.40	−0.04	

Temporally-defined global form	106	Step 1	0.03					
		Gender			0.29	1.13	0.03	
		SPM			−0.21	0.12	−0.17	
		Step 2	**0.13**⁎⁎	**0.10**⁎⁎				
		Gender			0.52	1.08	0.04	
		SPM			−0.15	0.11	−0.13	
		Reading skill			**−1.78**	**0.53**	**−0.31**⁎⁎	**0.11**

SPM = Raven’s Standard Progressive Matrices.

⁎ *p* < 0.05.

⁎⁎ *p* < 0.01.

⁎⁎⁎ *p* < 0.001.

**Table 5:** Group coherence threshold (%) statistics.

	Male (*N* = 33)						Female (*N* = 53)					
	Dyslexia (*N* = 17)			Good (*N* = 16)			Dyslexia (*N* = 26)			Good (*N* = 27)		
	*M*	*Median*	*SD*	*M*	*Median*	*SD*	*M*	*Median*	*SD*	*M*	*Median*	*SD*
Random-dot global motion	16.61	16.35	5.91	15.61	15.26	5.27	27.77	25.38	12.76	18.84	17.12	7.68
Spatially 1-D global motion	21.72	15.20	12.98	14.60	12.33	8.94	21.00	16.93	14.68	17.41	15.27	9.59
Static global form	14.37	14.93	3.57	15.49	13.81	5.70	15.06	15.15	3.30	14.78	13.15	4.58
Temporally-defined global form	93.49	93.39	4.09	88.33	89.77	7.89	92.75	93.92	5.05	90.76	92.67	5.89

**Table 6:** *Regression analyses: Between-group.* A model was run for each visual task with threshold as the dependent variable. The control variables (i.e. Gender and Non-Verbal IQ) were entered into the models at step one. Reading Group (Good = 0; Dyslexia = 1) was introduced at step two. Statistically significant results are shown in bold font.

Task	*N*	Step	*R*^2^	Δ*R*^2^	*B*	SE B	β	Cohen’s *f*^2^
Random-dot global motion	86	Step 1	**0.19**⁎⁎⁎					
		Gender			**6.63**	**2.04**	**0.32**⁎⁎	**0.12**
		SPM			**−0.57**	**0.21**	**−0.27**⁎⁎	**0.09**
		Step 2	**0.27**⁎⁎⁎	**0.08**⁎⁎				
		Gender			**6.80**	**1.96**	**0.33**⁎⁎⁎	
		SPM			**−0.52**	**0.20**	**−0.25**⁎	
		Reading group			**5.47**	**1.91**	**0.27**⁎⁎	**0.11**

Spatially 1-D global motion	86	Step 1	0.06					
		Gender			0.42	2.63	0.02	
		SPM			**−0.59**	**0.27**	**−0.24**⁎	**0.06**
		Step 2	**0.09**⁎	0.03				
		Gender			0.56	2.59	0.02	
		SPM			**−0.55**	**0.26**	**−0.22**⁎	
		Reading group			4.51	2.52	0.19	

Static global form	86	Step 1	0.00					
		Gender			−0.03	0.95	−0.00	
		SPM			−0.04	0.10	−0.05	
		Step 2	0.00	0.00				
		Gender			−0.04	0.96	−0.00	
		SPM			−0.05	0.10	−0.05	
		Reading group			−0.29	0.93	−0.03	

Temporally-defined global form	86	Step 1	0.03					
		Gender			0.57	1.32	0.05	
		SPM			−0.22	0.13	−0.18	
		Step 2	**0.10**⁎	**0.07**⁎				
		Gender			0.67	1.28	0.05	
		SPM			−0.19	0.13	−0.16	
		Reading group			**3.06**	**1.25**	**0.26**⁎	**0.07**

SPM = Raven’s Standard Progressive Matrices.

⁎ *p* < 0.05.

⁎⁎ *p* < 0.01.

⁎⁎⁎ *p* < 0.001.

## Footnotes

1 It should be noted that the sample, which comprised University Students is unlikely to capture the full range of reading skill variation in the population. Hence, the effect sizes reported here are likely to be conservative and might be greater in the general population. An advantage is that other factors known to be associated with reading skill, such as IQ, are similar, so the results are likely to reflect genuine differences in visual processing in relation to reading ability.
